# Cluster-based computational methods for mass univariate analyses of event-related brain potentials/fields: A simulation study

**DOI:** 10.1016/j.jneumeth.2014.08.003

**Published:** 2015-07-30

**Authors:** C.R. Pernet, M. Latinus, T.E. Nichols, G.A. Rousselet

**Affiliations:** a Centre for Clinical Brain Sciences, Neuroimaging Sciences, University of Edinburgh, Edinburgh, UK; b Institut de Neurosciences de la Timone UMR 7289, Aix Marseille Université, CNRS, 13385 Marseille, France; c Department of Statistics, Warwick University, Coventry, UK; d Institute of Neuroscience and Psychology, University of Glasgow, Glasgow, UK

**Keywords:** ERP, Family-wise error rate, Multiple comparison correction, Cluster-based statistics, Threshold free cluster enhancement, Monte-Carlo simulations

## Abstract

**Background:**

In recent years, analyses of event related potentials/fields have moved from the selection of a few components and peaks to a mass-univariate approach in which the whole data space is analyzed. Such extensive testing increases the number of false positives and correction for multiple comparisons is needed.

**Method:**

Here we review all cluster-based correction for multiple comparison methods (cluster-height, cluster-size, cluster-mass, and threshold free cluster enhancement – TFCE), in conjunction with two computational approaches (permutation and bootstrap).

**Results:**

Data driven Monte-Carlo simulations comparing two conditions within subjects (two sample Student's t-test) showed that, on average, all cluster-based methods using permutation or bootstrap alike control well the family-wise error rate (FWER), with a few caveats.

**Conclusions:**

(i) A minimum of 800 iterations are necessary to obtain stable results; (ii) below 50 trials, bootstrap methods are too conservative; (iii) for low critical family-wise error rates (e.g. *p* = 1%), permutations can be too liberal; (iv) TFCE controls best the type 1 error rate with an attenuated extent parameter (i.e. power < 1).

## 1 Introduction

Event-related potentials (ERP) and magnetic fields (ERF) are measurable cortical responses to events used to track cognitive processes. In a given experiment, they are observable at multiple locations in space (electrodes or magnetic field sensors) and time. ERP and ERF are characterized by various components which are stereotypic features such as a peak or trough at particular latencies.^1^ While for decades researchers have focused on analyzing such specific components, recent tools have been developed to analyze simultaneously the whole data space using a mass-univariate approach, whereby statistical tests are performed at every location and time point (e.g. Kiebel and Friston, 2004; Oostenveld et al., 2011; Pernet et al., 2011). This approach has the merit of not choosing locations or components a priori and therefore allows to potentially observing non-expected effects. Because so many statistical tests are performed, such approach can dramatically increase the odds of obtaining significant effects, i.e. there is a high probability of false positive results (type 1 error rate). Fortunately, different methods exist to control the family-wise error rate (FWER), i.e. the type 1 error rate over an ensemble, or family, of tests. The type 1 FWER is defined as the probability to make at least one type 1 error over the family of tests. Probably the best known method to control the FWER is the Bonferroni correction (Dunn, 1961) for which the alpha level is simply adjusted for the number of tests. This method is however overly conservative in the context of ERP/ERF analyses because it assumes statistical independence of the tests. For ERP and ERF, there are a large number of dependencies in space and in time, such that statistical tests are not independent. Methods used to control the type 1 FWER in such context must therefore account for these spatiotemporal dependencies.

ERP and ERF are distributed signals. Because there are a priori effects everywhere, it is common practice to discretize the data space and define treatment effects. Such discretization leads to the examination of treatment effects in terms of topological features like the maximum (e.g. +2 μV) or the extent (e.g. from +120 ms to +190 ms post stimulus onset) of the effect. In turn, this data reduction diminishes the multiple comparisons problem, while accounting for spatiotemporal dependences. One popular method that deals with multiple comparisons by taking into account the topology of the effects is random field theory (Worsley and Friston, 1995). Although it was developed for Positron Emission Tomography (PET) and functional Magnetic Resonance Imaging (fMRI), it has also been successfully applied to ElectroEncephaloGraphy (EEG) and MagnetoEncephaloGraphy (MEG) data (Kilner et al., 2005). Similarly, extensions of the Bonferroni correction for dependent data have been proposed (Hochberg, 1988). There are currently no large simulation results on the application of these methods to ERP/ERF, but previous work on real and simulated fMRI statistical images suggests that they are too conservative (Nichols and Hayasaka, 2003). In addition those methods also rely on various assumptions like positive dependence or smoothness. Here we choose to review alternative methods that combine cluster-based inference with assumption-free techniques like permutation, which have been shown to outperform analytic techniques to control the type 1 FWER (Nichols and Hayasaka, 2003).

**Fig. 1:**
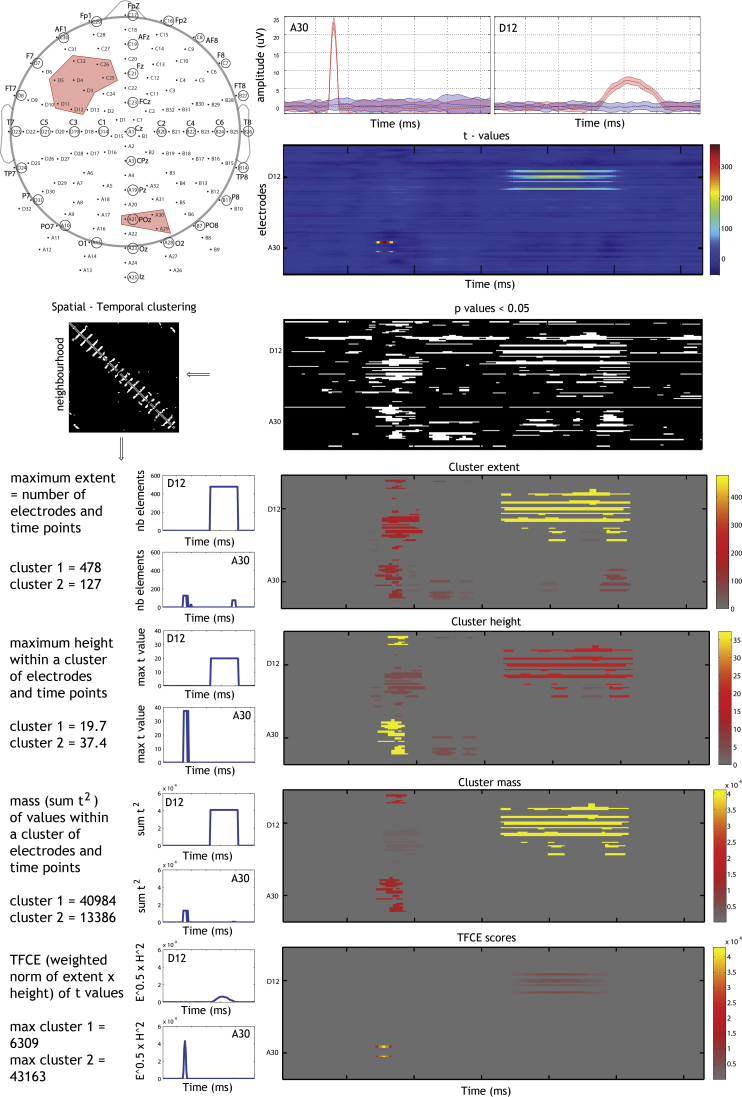
Illustration of cluster-based methods applied to caricatured ERP data. Two effects were created, one transient effect (+25 μV) over 3 right posterior electrodes and one more sustained effect (+7 μV) over 8 electrodes. These effects are not meant to represent true EEG signal, but illustrate the different cluster attributes that are obtained on the basis of thresholded *t* values. From the observed *t* values, a binary ‘map’ is obtained (i.e. *p* < 0.05), and cluster attributes and TFCE data are computed via spatiotemporal clustering (3 first rows of the figure). The transformed data, to be thresholded, are presented for 2 electrodes (D12 and A30) and over the full space. Because the statistics are now based on cluster attributes, effect sizes can differ substantially from the original effects: (i) with cluster extent, effect-sizes are reversed with the sustained effect being stronger than the transient effect because it has a large support in space and time; (ii) cluster-height preserves effect-sizes but discards spatiotemporal information; (iii) with cluster-mass, effect-sizes are reversed but the difference between the sustained effect and the transient effect is attenuated compared to cluster-extend because cluster-mass accounts for height; (iv) with TFCE effect-sizes are preserved, and in contrast to cluster attributes, the shape of each effect is also preserved.

Cluster-based statistics consist in grouping together neighboring variables (*t* or *F* values for instance) into clusters and deriving characteristic values for the clusters. Typically, a cluster is characterized by its height (maximal value), its extent (number of elements) or a combination of both (Poline and Mazoyer, 1993). In the last case, this is often obtained by summing the statistical values within a cluster, an approach referred to as cluster-mass (Bullmore et al., 1999; Maris and Oostenveld, 2007 – see Fig. 1 for an illustration). A first issue with traditional cluster inference is that clusters need to be defined by setting a ‘cluster forming threshold’ (Gorgolewski et al., 2012). In practice, statistical values are considered for inclusion in a cluster only if they are higher than the cluster forming threshold, for instance a univariate *p* < 0.05. It is then possible to compute clusters’ attributes and their associated probabilities. A second issue with cluster statistics is that inferences are limited to clusters, i.e. one cannot be certain of the significance of single elements inside clusters. More recently, Smith and Nichols (2009) have proposed to ‘enhance’ *t* or *F* values, by integrating attributes (height and extent) computed for all possible a priori cluster forming thresholds (Eq. (1)), leading to statistical maps where each data point, rather than cluster, can be thresholded. This method, referred to as Threshold Free Cluster Enhancement (TFCE), has the advantage of alleviating issues of setting a cluster forming threshold and of cluster inference and has been shown to control the type 1 FWER for ERP using permutation (Mensen and Khatami, 2013).

(1) $$\text{TFCE}\;(\text{loc,time})=\int_{h=h0}^{h(\text{loc,time})}\text{extent}\;{\left(h\right)}^{E}\text{height}\;{\left(h\right)}^{H}dh$$

The TFCE value at a given location (loc) and time point (time) is the integral of all cluster-extents × cluster-heights from *h*0 (typically the minimum value in the data) to *h* (typically the maximum value in the data). Parameters *E* and *H* are set to 0.5 and 2 respectively in LIMO EEG (these choices are discussed below). In practice the integral is estimated as a sum, using finite *dh* (here *dh* = 0.1). As discussed by Smith and Nichols (2009), TFCE is a generalization of the cluster-mass statistic (*E* = 1, *H* = 0), and can be related to Random Field Theory cluster *p*-values.

In the present study, using data driven simulations, we evaluated the ability of cluster-based computational methods to control the type 1 FWER. The first goal of the study was to establish the equivalence of permutation and bootstrap procedures to control the type 1 FWER, in the context of cluster-mass for ERP. Cluster-mass is the method implemented in both LIMO EEG (Pernet et al., 2011; https://gforge.dcn.ed.ac.uk/gf/project/limo_eeg/) and FieldTrip (Maris and Oostenveld, 2007; http://fieldtrip.fcdonders.nl/). LIMO EEG uses a bootstrap-*t* technique, and FieldTrip uses permutation, but the two techniques have not been compared in this context. In addition, cluster-mass was only validated, in FieldTrip, for time–frequency data. The second goal of this study was to validate the TFCE method for ERP, using the bootstrap-*t* technique implemented in LIMO EEG, as opposed to permutation as in Mensen and Khatami (2013).

## 2 Methods

Codes used to generate and analyze the data are available on FigShare at http://figshare.com/articles/Type_1_error_rate_using_clustering_for_ERP/1008311. The data generated to compare clustering approaches are too large to be shared (∼32 GB) but the code could be used on any data and similar results are expected. Intermediate results (i.e. FWER data per subject) are nevertheless available. For the TFCE simulations, codes and intermediate data are also available at http://figshare.com/articles/Type_1_error_rate_using_clustering_for_ERP/1008325.

### 2.1 Cluster-attributes using bootstrap and permutation

Whilst simulations aimed at comparing bootstrap and permutation techniques in the context of cluster-mass, we also computed cluster-extent and cluster-height to enquire potential differences. Simulations were performed in the context of a within subject two sample Student's t-test, comparing two hypothetical conditions, but results apply in principle to other within subject cases.

Ten subjects of the LIMO EEG dataset (Rousselet et al., 2009; Rousselet et al., 2010) were randomly chosen as representative ERP data. Data were from a 128 electrodes Biosemi system, 250 Hz sampling rate, with 201 time point epochs ranging from −300 ms to 500 ms. During recording, subjects discriminated between two faces with various levels of noise (see references for details) and performed over 1000 trials. One thousand Monte Carlo (MC) simulations were performed per subject (10,000 MC in total). For each Monte Carlo and each subject, samples of 10, 25, 50, 100, 300, 500, 900 trials per groups were obtained by increment, e.g. when *N* = 25, the same 10 trials as with *N* = 10 were present. For each MC, data were randomly assigned to a condition (say face A versus face B) and a two sample Student's t-test computed for every electrode and time point. Three techniques were used to estimate the null hypothesis (H0) for the sample considered: (i) a *permutation t-test* in which all the trials from the two conditions are permuted randomly between conditions and a *t*-test is computed for each permutation, (ii) a modified *percentile bootstrap* in which all the trials from the two conditions are first pooled together, then sampled with replacement and randomly assigned to the two conditions and a *t*-test computed for each bootstrap, (iii) a *bootstrap-t* in which each condition is first mean centered, then sampled with replacement and a *t*-test is computed for each bootstrap. For all three techniques, 1000 iterations were computed and maximal values recorded using cluster-forming thresholds of *p* = 0.05 and *p* = 0.01. In total 7000 draws of data were computed per subject (1000 MC × 7 sample sizes) and 42,000 statistical maps obtained (7000 draws × 3 techniques × 2 cluster-forming thresholds).

#### 2.1.1 Exploratory data analysis

For each subject, technique, cluster-forming threshold, and sample size, we computed the null distributions of maxima for the 3 cluster statistics: cluster-height, cluster extent, and *t*^2^ cluster-mass and thresholded the sampled data according to these distributions. The type 1 FWER for the critical FWE thresholds of *p* = 0.05 and *p* = 0.01 was computed as the probability to obtain at least one significant effect across all electrodes and time points over the 1000 MC and then averaged over subjects. Deviation from the set type 1 error rate was tested for each combination of cluster statistics and technique (e.g. cluster-mass/permutations) by computing percentile bootstrap 95% confidence intervals (CI) with a Bonferroni adjustment for simultaneous probability coverage over the 7 sample sizes (i.e. alpha = 0.9929% – Wilcox, 2012). We also compared the percentage of agreement between the different techniques, which is the proportion of times the same results were observed out of 10 × 1000 MC. Finally, we looked at how many permutations or bootstraps were necessary (from 200 to 1000 by steps of 200) to achieve the nominal FWER.

#### 2.1.2 Confirmatory data analysis

To test the equivalence of permutation and the two bootstrap procedures for cluster-mass inference, simulation results were collapsed over all sample sizes and a percentile bootstrap (with a Bonferroni adjustment for multiple comparisons) was computed on the mean, 20% trimmed mean, and median type 1 FWER, testing if the nominal level was obtained. Pair-wise comparisons of the different techniques (permutation vs. percentile bootstrap, permutation vs. bootstrap t and percentile bootstrap vs. bootstrap *t*) were also performed using a percentile bootstrap on the mean differences.

### 2.2 TFCE validation

A different set of data driven Monte Carlo simulations were performed on the same 10 subjects as above. For each subject, 1000 MC samples of 200 trials, 100 trials per condition, were drawn randomly and with replacement from a pool of over 1000 trials, thus mimicking a series of draws from the same population. For each MC sample, a two-sample Student's *t*-test was computed. For each of these *t*-tests, the null hypothesis was evaluated using a bootstrap-*t* technique with 1000 iterations. The observed *t* values were thresholded using cluster-mass (cluster-forming threshold *p* = 0.05) and using TFCE. Since TFCE integrates multiple thresholded maps, the cluster extent and height can have different powers leading to different enhanced values (Eq. (1)). Here we tested 4 possible combinations of TFCE parameters: extent^0.5*height^1, extent^0.5*height^2, extent^1*height^1 and extent^1* height^2. For each subject, the 5000 maps (1000 MC for cluster-mass + 1000 MC × 4 TFCE) were thresholded at a critical 5% FWE threshold and the type 1 FWER computed. The mean, 20% trimmed mean, and median FWER across subjects for the 4 combinations of TFCE parameters were computed and tested against the nominal level (percentile bootstrap with alpha adjusted for simultaneous probability coverage over the 4 possible combinations) and compared with cluster-mass (bootstrap-*t* with alpha adjusted for multiple comparisons).

## 3 Results

### 3.1 Exploratory data analysis

**Fig. 2:**
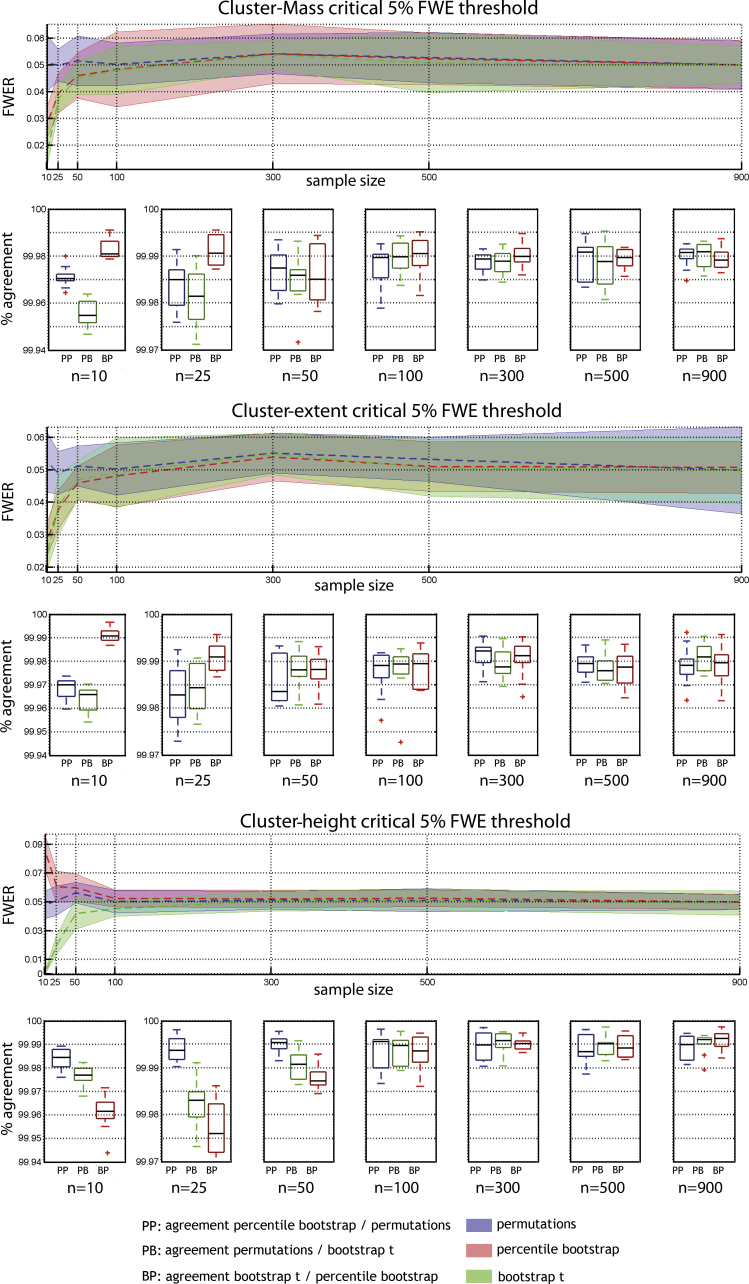
Type 1 FWER and percentages of agreement for a critical 5% FWE (cluster forming threshold *p* = 0.05). Results are presented per cluster statistic with curves showing the mean FWER across subjects with adjusted 95% confidence intervals. Boxplots show the median and inter-quartile range agreement between techniques (outliers marked with plus signs).

**Fig. 3:**
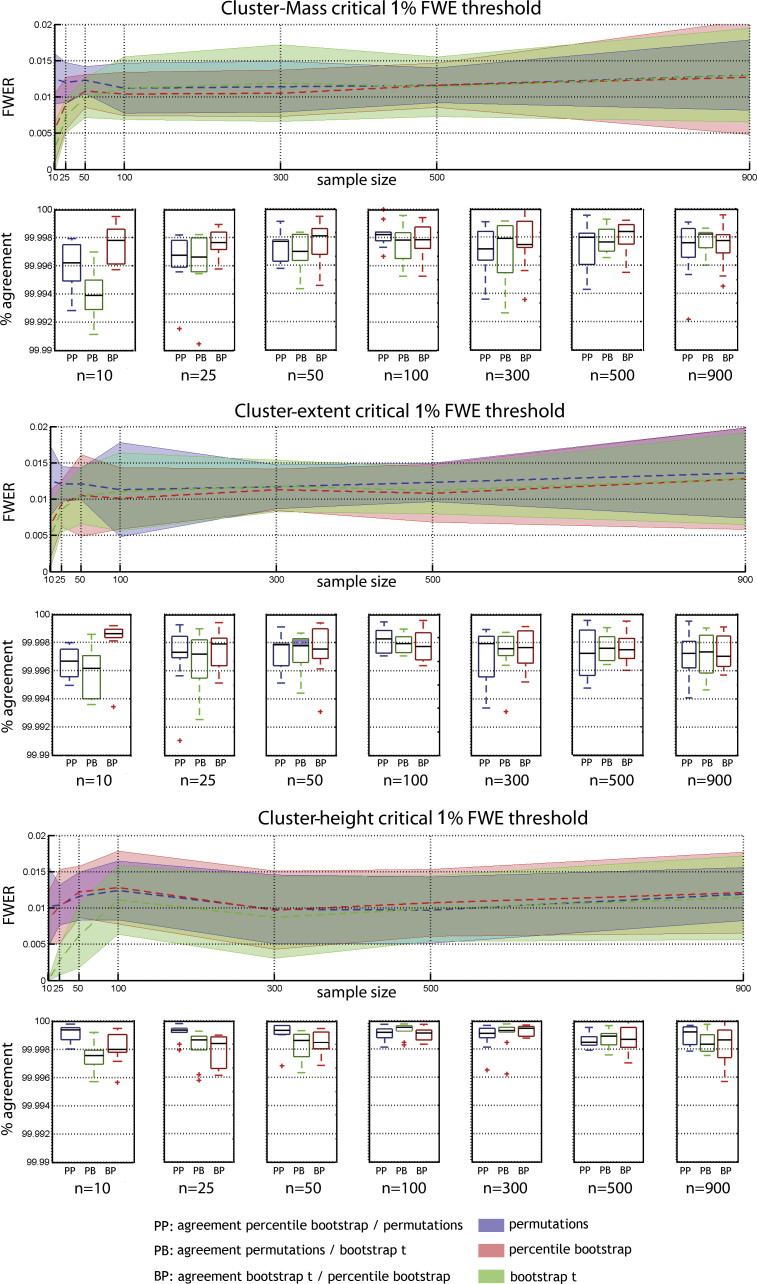
Type 1 FWER and percentages of agreement for a critical 1% FWE (cluster forming threshold *p* = 0.01). Results are presented per cluster statistic with curves showing the mean FWER across subjects with adjusted 95% confidence intervals. Boxplots show the median and inter-quartile range agreement between techniques (outliers marked with plus signs).

**Fig. 4:**
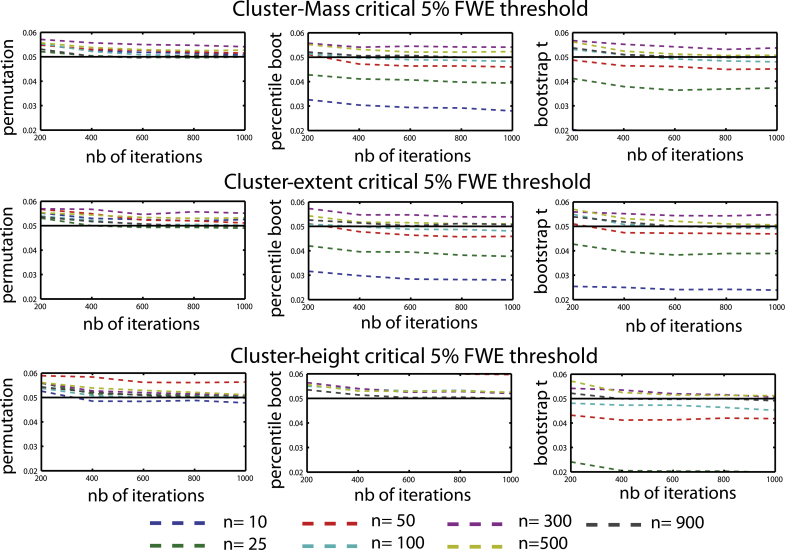
Type 1 FWER for a critical 5% FWE (cluster forming threshold *p* = 0.05) for each cluster statistic and technique as a function of the number of sampling iterations for the 7 sample sizes tested (*n* = [10 25 50 100 300 500 900] per group).

**Fig. 5:**
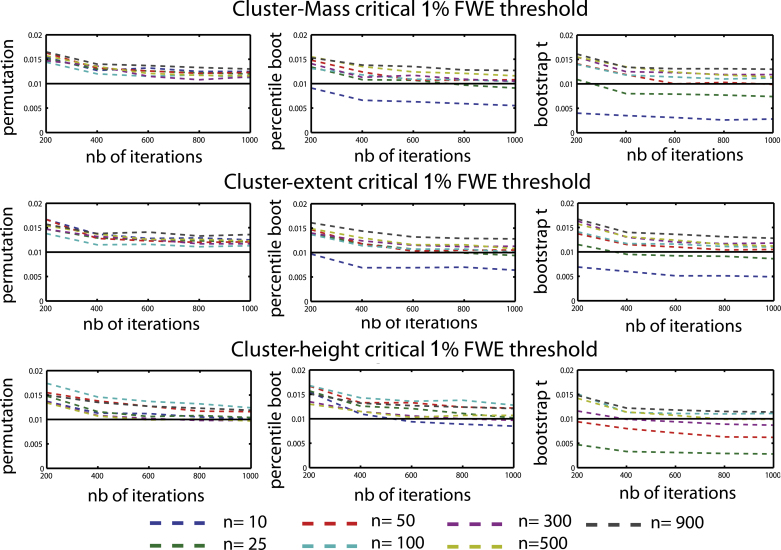
Type 1 FWER observed for a critical 1% FWE (cluster forming threshold *p* = 0.01) for each cluster statistic and technique as a function of the number of sampling iterations for the 7 sample sizes tested (*n* = [10 25 50 100 300 500 900] per group).

Results show that the six combinations of techniques (permutation, bootstrap) and cluster statistics (cluster-mass, cluster-extent, cluster-height) controlled well the type 1 FWER (Figs. 2 and 3). In addition, we observed a high percentage of agreement between statistical masks (always >99%) demonstrating the equivalence of the techniques. The only cases where the type 1 FWER deviated from the nominal level was with the smallest sample sizes (*N* = 10 or 25 trials per condition), where bootstraps were too conservative. One exception was observed for cluster-height under percentile bootstrap with a critical 5% FWER, which gave a too high value. To achieve the nominal FWER (Figs. 4 and 5), between 600 and 800 iterations were necessary, irrespective of the technique considered. If fewer than 600 iterations were drawn, results were too liberal (except again for bootstrap techniques with small sample sizes which were always too conservative).

### 3.2 Confirmatory data analysis

For a critical 5% FWER, on average across sample sizes, cluster-mass inference for the three techniques gives a FWER that did not differ significantly from 5% (Table 1). Significant mean differences were nevertheless observed among the three techniques, with permutation showing systematically higher FWER than bootstrap: permutation vs. percentile bootstrap 0.0016 [0.0004, 0.0027] *p* = 0; permutation vs. bootstrap-*t* 0.0022 [0.0011, 0.0035] *p* = 0; percentile bootstrap vs. bootstrap-*t* 0.0006 [−0.0003, 0.0015] *p* = 0.09.

For a critical 1% FWER, on average across sample sizes, cluster-mass inference for the three techniques gives a FWER close to the nominal level, with significant deviation in the case of permutations, being too liberal (Table 2). The mean FWER was significantly higher for permutations compared to the percentile bootstrap (0.007 [0.0001, 0.0012] *p* = 0.006), but not to the bootstrap-*t* (0.0042 [−0.0002, 0.001] *p* = 0.09). The percentile bootstrap did not differ significantly from bootstrap-*t* (−0.0028 [−0.0009, 0.0003] *p* = 0.34).

### 3.3 TFCE validation

**Fig. 6:**
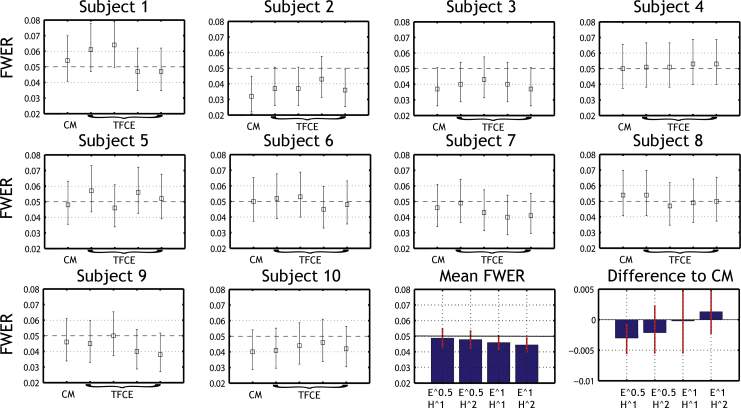
Type 1 FWER for cluster-mass (CM) and Threshold Free Cluster Enhancement (TFCE) using 4 combinations of extent and height. Boxes show for each subject the mean type 1 FWER and associated binomial 95% CI. The bottom right plots show bar graphs of the mean type 1 FWER across subjects and 95% CI, and the mean differences between each TFCE parameter set and cluster-mass type 1 FWER.

The percentile bootstrap *t*-test with simultaneous 95% probability coverage showed that TCFE with the extent parameter of power 1 was too conservative (Table 3, Fig. 6). Comparisons with cluster-mass show that the parameter combination extent^0.5*height^1 was significantly less conservative than cluster-mass, while other combinations show results similar to cluster-mass.

## 4 Discussion

Overall, our simulations show that cluster-based approaches provide a type 1 FWER close to or at the nominal level. Three exceptions were nevertheless observed: (i) cluster-statistics with permutation can to be too liberal; (ii) cluster-statistics with bootstrap techniques and sample sizes of 10 and 25 per group are too conservative; (iii) TFCE with extent parameters at power 1 are too conservative. With regards to the study main goals we showed that (i) permutation and bootstrap techniques give, on average, very similar results and that (ii) TFCE, in conjunction with bootstrap, can be a valid method for ERP inference.

While some deviations are expected between techniques, bootstrap showed strong deviations for small sample sizes, which can be explained by the high cluster statistic values obtained under H0. To illustrate, let us consider cluster-mass with a critical 5% FWER threshold and *N* = 10: over the 10 × 1000 Monte Carlo simulations, permutation gave a range of cluster-mass thresholds from 1390 to 24,300 (median 18,321), whereas the percentile bootstrap gave a range of cluster-mass thresholds from 17,843 to 31,594 (median 22,954), and the bootstrap-*t* a range of cluster-mass thresholds from 21,096 to 44,036 (median 29,190). One reason for the elevated bootstrap thresholds could relate to the sampling scheme. A *t*-test is defined by the ratio between the mean difference and the square root of the sum of standardized variances. During bootstrap resampling, if only few unique trials are drawn, this can reduce the variance to a point where the denominator is inferior to 1, which in turn can lead to large *t* values. In the simulations, we did not constraint the number of unique trials, and re-running the sampling method to generate indices indicates that, for a given subject, no unique values were drawn, but as low as 2 unique trials were used which would have led to high *t* values. Permutation is not affected by this issue because the same number of different trials is present at each iteration, maintaining variance at a reasonable level. For small sample sizes, it is thus recommended to use a permutation test, or to constraint the minimum number of unique observations in the bootstrap samples. Of course, whatever technique is used, statistical inferences are fundamentally limited when only 10 trials are used.

All techniques tended to have the same rate of convergence, with a flattening of the type 1 FWER curves after ∼600 iterations (Figs. 4 and 5). Troendle et al. (2004) showed that bootstrap procedures can be too conservative when using maximum *t* statistic in a multivariate context, especially with small sample sizes. Our results suggest that for maximum cluster-statistics, results are stable after 600–800 iterations, and any observed variations in the type 1 FWER are due to small sample sizes when using bootstrap approaches. Also, there does not seem to be any advantage in using more than 800 iterations: in one subject, 1000 MC using a bootstrap-*t* test with *N* = 10 showed no changes in type 1 error rate between 800 iterations and up to 3000 iterations (type 1 FWER = 0.019).

We validated the use of TFCE in conjunction with a bootstrap-*t* technique in the context of ERP analyses, and showed that using *E* = 0.5 (i.e. extent^0.5 in Eq. (1)), the type 1 error rate is at the nominal level. Using *E* = 1 gave too conservative results. Under the null hypothesis, this is likely due to large clusters when *h* is small, which, once integrated lead to high TFCE thresholds, which in turn lead to a conservative type 1 FWER. As pointed out by Smith and Nichols (2009), at the lowest values of *h*, the significant clusters are too large and do not provide very useful spatiotemporal specificity, and it is therefore preferable to scale down their effect. Conversely, for the parameter *H*, when *H* > 1, the TFCE scores scale supra-linearly with increasing statistic image intensity, which can be consider to be desirable. We thus follow Smith and Nichols and also suggest to use *H* = 2 (i.e. height^2 in Eq. (1)), since squaring follows the log of *p*-values (see Smith and Nichols, 2009) and gives results similar to cluster-mass in our simulations. Mensen and Khatami (2013) validated TFCE for EEG using permutation, with parameters *E* = 2/3, *E* = 1 and *H* = 2. In their simulations the data had both no effect (H0) and some effects (H1), and they computed the balance between type 1 and type 2 error rates. The best result was obtain with *E* = 1. Given that they did not test the FWER over the whole space, we believe that *E* < 1 remains the best option to achieve the nominal FWER.

The type 1 FWER was estimated with real null data and therefore there are no issues of signal-to-noise ratio (SNR), or number of sources. In addition, because the methods described here adapt to the acquisition parameters by estimating the null hypotheses from the data themselves, aspects of data acquisition related to sampling (sampling rate or number of electrodes) should not affect the interpretation of our results. In this type of simulation, only the variance–covariance structure has an influence on the results. Although the large scale structure of the covariance matrix (i.e. which electrodes correlate with which, when) depends on the experimental set-up, the smaller scale structure of the covariance matrix (i.e. how trials correlate) is expected to be similar across datasets. We can therefore expect to obtain similar results using other datasets, and we do remind interested readers that the code is available to try on their own data. In contrast to the type 1 FWER tested here, many parameters will affect power. The ability to detect an effect and declare this effect as significant is likely to depend on the SNR, the spatiotemporal sampling, and the statistical method used. In particular, cluster extent and cluster height will be affected by differences in source depth and SNR and it is recommended to use cluster-mass or TFCE in most situations.

In conclusion we showed that permutation and bootstrap techniques can be used alike in combination with cluster statistics, including TFCE, providing accurate type 1 FWER. In the case of small sample sizes (*N* = 10, 25 trials), it is advisable to use permutation as it offers a better control over the type 1 FWER, whereas bootstrap techniques are too conservative. Conversely, for larger sample sizes (*N *≥ 50), it is advisable to use bootstrap techniques because permutation can be too liberal. Finally, although our simulations aimed to show generally applicable results, it remains to be tested how these techniques behave with data having different variance structures. For instance, between subject analyses tend to have small samples with large variances, which can be a problem for permutation if the data are heteroscedastic (Efron and Tibshirani, 1993).

## Figures and Tables

**Table 1:** Mean, 20% trimmed mean and median of the cluster-mass FWER for a 5% critical threshold. In brackets are the adjusted 95% CI.

	Permutation	Percentile bootstrap	Bootstrap-*t*
Mean	0.0517 [0.0498, 0.0536]	0.0501 [0.0479, 0.0523]	0.0495 [0.0471, 0.0519]
20% Trimmed mean	0.0517 [0.0497, 0.0540]	0.0497 [0.0476, 0.0524]	0.0493 [0.0469, 0.0519]
Median	0.0515 [0.049, 0.054]	0.0495 [0.048, 0.0515]	0.049 [0.047, 0.0515]

**Table 2:** Mean, 20% trimmed mean and median of the cluster-mass FWER for a 1% critical threshold. In brackets are the adjusted 95% CI. Significant deviations from the nominal level are in bold.

	Permutation	Percentile bootstrap	Bootstrap *t*
Mean	**0.0119 [0.0109,0.0129]**	0.0112 [0.0100, 0.0124]	**0.0115 [0.0102,0.0127**]
20% Trimmed mean	**0.0119 [0.0110,0.013]**	0.0109 [0.0099, 0.012]	0.0115 [0.0099, 0.0131
Median	**0.0120 [0.011,0.013]**	0.0110 [0.009, 0.012]	0.0110 [0.01, 0.013]

**Table 3:** TFCE mean type 1 FWER (critical 5% FWER) and mean differences between TFCE and cluster-mass. The adjusted 95% CI are indicated in square brackets, and significant deviations are in bold.

	Mean FWER	Difference to cluster-mass
Cluster-mass	0.0457 [0.0392, 0.0522]	
TFCE *E*^0.5*H^1	0.0487 [0.0435, 0.0539]	**−0.0030 [−0.0053, −0.0008]**
TFCE *E*^0.5*H^2	0.0478 [0.0423, 0.0533]	−0.0021 [−0.0057, 0.0017]
TFCE *E*^1*H^1	**0.0459 [0.0423, 0.0495]**	−0.0002 [−0.0058, 0.0044]
TFCE *E*^1*H^2	**0.0444 [0.0403, 0.0485]**	0.0013 [−0.0020, 0.0045]

## Footnotes

1 http://www.sinauer.com/fmri2e/html/glossary.html.
